# Admix-kit: an integrated toolkit and pipeline for genetic analyses of admixed populations

**DOI:** 10.1093/bioinformatics/btae148

**Published:** 2024-03-15

**Authors:** Kangcheng Hou, Stephanie Gogarten, Joohyun Kim, Xing Hua, Julie-Alexia Dias, Quan Sun, Ying Wang, Taotao Tan, Polygenic Risk Methods in Diverse Populations (PRIMED) Consortium Methods Working Group, Sally Adebamowo, Adebowale Adeyemo, Paul Auer, Taoufik Bensellak, Sonja Berndt, Rohan Bhukar, Hongyuan Cao, Clinton Cario, Nilanjan Chatterjee, Jiawen Chen, Tinashe Chikowore, Ananyo Choudhury, Matthew Conomos, David Conti, Sinead Cullina, Burcu Darst, Yi Ding, Ruocheng Dong, Rui Duan, Yasmina Fakim, Nora Franceschini, Tian Ge, Anisah W Ghoorah, Chris Gignoux, Stephanie Gogarten, Neil Hanchard, Rachel Hanisch, Michael Hauser, Scott Hazelhurst, Jibril Hirbo, Whitney Hornsby, Kangcheng Hou, Xing Hua, Alicia Huerta, Micah Hysong, Jin Jin, Angad Johar, Jon Judd, Linda Kachuri, Abram Bunya Kamiza, Eimear Kenny, Alyna Khan, Elena Kharitonova, Joohyun Kim, Iain Konigsberg, Charles Kooperberg, Matt Kosel, Iftikhar Kullo, Ethan Lange, Yun Li, Qing Li, Maria Liivrand, Kirk Lohmueller, Kevin Lu, Ravi Mandla, Alisa Manning, Iman Martin, Alicia Martin, Shannon McDonnell, Leah Mechanic, Josep Mercader, Rachel Mester, Maggie Ng, Kevin Nguyen, Kristján Norland, Franklin Ockerman, Loes Olde Loohuis, Ebuka Onyenobi, Bogdan Pasaniuc, Aniruddh Patel, Ella Petter, Kenneth Rice, Joseph Rothstein, Bryce Rowan, Robb Rowley, Yunfeng Ruan, Sriram Sankararaman, Ambra Sartori, Dan Schaid, Ruhollah Shemirani, Jonathan Shortt, Xueling Sim, Johanna L Smith, Maggie Stanislawski, Daniel Stram, Quan Sun, Bamidele Tayo, Buu Truong, Kristin Tsuo, Sarah Urbut, Ying Wang, Wallace Minxian Wang, Riley Wilson, John Witte, Genevieve Wojcik, Jingning Zhang, Ruyue Zhang, Haoyu Zhang, Yuji Zhang, Michael Zhong, Laura Zhou, Elizabeth G Atkinson, Alicia Martin, Jonathan Shortt, Jibril Hirbo, Yun Li, Bogdan Pasaniuc, Haoyu Zhang

**Affiliations:** Bioinformatics Interdepartmental Program, University of California, Los Angeles, Los Angeles, CA, 90095, United States; Department of Biostatistics, University of Washington, Seattle, WA, 98195, United States; Vanderbilt Genetics Institute and Division of Genetic Medicine, Vanderbilt University Medical Center, Nashville, TN, 37232, United States; Division of Cancer Epidemiology and Genetics, National Cancer Institute, Bethesda, MD, 20892, United States; Department of Biostatistics, Harvard T.H. Chan School of Public Health, Boston, MA, 02120, United States; Department of Biostatistics, University of North Carolina at Chapel Hill, Chapel Hill, NC, 27599, United States; Program in Medical and Population Genetics, Broad Institute of MIT and Harvard, Cambridge, MA, 02142, United States; Department of Molecular and Human Genetics, Baylor College of Medicine, Houston, TX, 77030, United States; Department of Molecular and Human Genetics, Baylor College of Medicine, Houston, TX, 77030, United States; Program in Medical and Population Genetics, Broad Institute of MIT and Harvard, Cambridge, MA, 02142, United States; Department of Biomedical Informatics, University of Colorado Anschutz Medical Campus, Aurora, CO, 80045, United States; Division of Genetic Medicine, Department of Medicine, Vanderbilt University Medical Center, Nashville, TN, 37232, United States; Department of Biostatistics, University of North Carolina at Chapel Hill, Chapel Hill, NC, 27599, United States; Bioinformatics Interdepartmental Program, University of California, Los Angeles, Los Angeles, CA, 90095, United States; Division of Cancer Epidemiology and Genetics, National Cancer Institute, Bethesda, MD, 20892, United States

## Abstract

**Summary:**

Admixed populations, with their unique and diverse genetic backgrounds, are often underrepresented in genetic studies. This oversight not only limits our understanding but also exacerbates existing health disparities. One major barrier has been the lack of efficient tools tailored for the special challenges of genetic studies of admixed populations. Here, we present admix-kit, an integrated toolkit and pipeline for genetic analyses of admixed populations. Admix-kit implements a suite of methods to facilitate genotype and phenotype simulation, association testing, genetic architecture inference, and polygenic scoring in admixed populations.

**Availability and implementation:**

Admix-kit package is open-source and available at https://github.com/KangchengHou/admix-kit. Additionally, users can use the pipeline designed for admixed genotype simulation available at https://github.com/UW-GAC/admix-kit_workflow.

## 1 Introduction

Admixed individuals inherit a mosaic of ancestry segments originating from multiple continental ancestral populations, leading to their complex and diverse genetic backgrounds encompassing a wide spectrum of human genetic variation (Seldin *et al.* 2011). Admixed individuals carry an elevated number of genetic variants in the 1000 Genomes Project (Auton *et al.* 2015). For example, African Americans contain genetic variants originating from both European and African ancestral populations, offering a unique opportunity to study genetic variation from multiple continental populations within a single population. Therefore, an understanding of such genetic ancestry mosaicism within admixed populations offers opportunities to gain insights into the origins and health implications of various genetic traits and diseases, contributing to a more comprehensive understanding of human genetics (Wojcik *et al.* 2019, Tan and Atkinson 2023).

Despite the genetic richness and crucial insights they can offer, admixed populations remain significantly underrepresented in current genetic studies (Mills and Rahal 2020). This underrepresentation can be attributed to various challenges, including the complexity of analyzing diverse genetic backgrounds and the lack of efficient tools and standardized practices for handling the genetic data of admixed populations. This gap not only hinders progress in genetic research but also exacerbates health disparities. For example, findings with datasets from European ancestry groups for genetic risk prediction models can introduce bias to personalized risk prevention strategies (Martin *et al.* 2019, Ding *et al.* 2023). Genetic admixture is key to understanding variations in phenotype and disease prevalence across populations (Gurdasani *et al.* 2019). A notable example is the lower white blood cell count observed in individuals of African ancestry (Reich *et al.* 2009). Such genetic differences, if overlooked, can lead to clinical misinterpretations and unnecessary procedures, including bone marrow biopsies (Van Driest *et al.* 2021).

To address these challenges, we introduce admix-kit, an integrated and flexible python toolkit along with workflows developed using Workflow Development Language (WDL), specifically designed for the simulation and analysis of genetic data from admixed populations. We anticipate that our proposed software packages and workflows will help overcome these analytical challenges, enabling the inclusion of admixed individuals in future genetic studies.

## 2 Results

### 2.1 Computational toolkit for analyzing admixed genotypes

**Figure 1.**
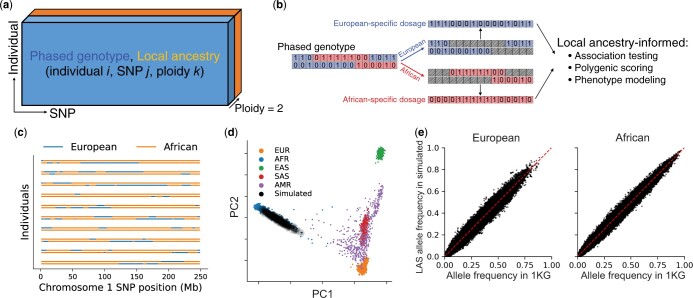
Overview of admix-kit’s data structure, functionality, and illustrative analyses using a simulated dataset. (a) Local ancestry and phased genotypes are stored in matrix format. Individual-specific and SNP-specific covariates are stored as two tables with matching orders. (b) Analysis based on ancestry-specific genotype dosage. Starting with a phased genotype for an individual (0/1 denotes presence of minor allele), genotypes are separated into ancestry-specific dosages. Local ancestry-informed downstream analyses can be subsequently performed. (c) visualization of local ancestry tracts. (d) Consistency of genome-wide genetic ancestry of simulated dataset using YRI and CEU in 1000 Genomes as reference populations. (e) Consistency of allele frequencies from the simulated admixed genotypes.

We begin by outlining the data structures and computational tools in admix-kit for analyzing admixed genetic datasets. Both genotype and local ancestry data are organized as two matrices of shape *N* × *M* × 2 (*N* and *M* denote the number of individuals and SNPs respectively, and ‘2’ denotes the two haplotypes; Fig. 1a). Given that storage of these matrices often exceeds memory capacity (due to large *N* and/or *M*), we adopt a chunked array representation, implemented with the Dask python library (Rocklin 2015). Each chunk is loaded from disk on demand, thus conserving memory by loading data only when needed and facilitating large-scale analyses. We use pgenlib as an efficient python interface to read phased genotype. Local ancestry matrices are stored in a compressed format that leverages their contiguous nature (local ancestries for nearby SNPs are often identical within each individual). By translating genotype and local ancestry matrices into local-ancestry-specific (LAS) genotype dosages, we have also implemented a set of utility functions tailored for LAS genetic analysis, including LAS allele frequencies, polygenic scores, and phenotype modeling that allow for LAS genetic architecture (Fig. 1b).

### 2.2 Workflow for simulating admixture genotypes

Genotype simulation is essential to facilitate testing and benchmarking genetic analysis methodologies. One of the significant challenges lies in simulating admixed genomes, which often becomes the most time-consuming step among common analyses involving admixture. We develop a workflow to specifically address this bottleneck (Supplementary Fig. S1a). We primarily focus on two-way admixture for demonstration while noting our software and pipeline are adaptable to various admixture scenarios. First, starting from a small reference panel such as 1000 Genomes Project, HAPGEN2 (Su *et al.* 2011) is used to enhance the diversity and size of reference dataset by increasing number of unique haplotypes via recombining initial sets of haplotypes within each genetic ancestry group, such as European or African. This step increases the reference data sample size while preserving the minor allele frequency (MAF) and linkage disequilibrium (LD) structure. Second, using the expanded haplotype sets in both genetic ancestry groups, we simulate admixture process using haptools (Massarat *et al.* 2023) with parameters for genetic ancestry proportion and the number of admixture generations. We are primarily interested in simulation scenarios involving an instantaneous admixture event, followed by generations of haplotype recombination (we note other scenarios such continuous admixture events can also be simulated). This process mimics random mating and recombination events to generate realistic distribution of local ancestry segments, MAF and LD structure for the generated genotypes. To make this simulation process more accessible, we have implemented these functionalities as command-line tools within admix-kit (Supplementary Fig. S1a). In details, admix hapgen2 --pfile ${src_plink2} --n-indiv ${n_indiv} --out ${expanded_pop} is used to expand the source population with HAPGEN2. And admix admix-simu --pfile-list "['pop1', 'pop2']" --admix-prop "[0.2,0.8]" --n-indiv ${n_indiv} --n-gen ${n_gen} --out ${admix} can be used to simulate the admixture process across source populations.

Furthermore, we included a number of functions to perform LAS genetic analysis. For genotype-phenotype association testing (admix assoc), we have implemented a suite of methods allowing for genetic effects heterogeneity across local ancestry backgrounds (Pasaniuc *et al.* 2011, Atkinson *et al.* 2021, Hou *et al.* 2021, Mester *et al.* 2023). These approaches can enhance statistical power by modeling LAS-genetic architecture within admixed populations. We also include functionality to estimate genetic effects concordance across local ancestry backgrounds (admix genet-cor), which is crucial to understand and inform trait-specific optimal strategies for downstream analyses including polygenic score weight estimation and application (Hou *et al.* 2023). For polygenic scoring (admix calc-partial-pgs), we implemented the calculation of partial polygenic scores, allowing scores to be computed separately for each ancestry background. Such an approach can improve polygenic scoring accuracy in admixed populations (Bitarello and Mathieson 2020, Marnetto *et al.* 2020, Cai *et al.* 2021, Sun *et al.* 2022). We also provided a user-friendly WDL-based workflow for genotype simulation that can be run on cloud-based computing platforms [e.g. AnVIL (https://anvilproject.org/), BioData Catalyst (https://biodatacatalyst.nhlbi.nih.gov/)] (Supplementary Fig. S1b). Users can input essential parameters to define the admixture process and provide the input genotype path of ancestral populations (a set of preprocessed 1000 genomes dataset is provided for default usage). The workflow will run through each aforementioned step and produce the simulated admixed genotype dataset. The admix-kit software is encapsulated in a publicly available docker image (URLs).

### 2.3 Example analysis of a simulated dataset

We demonstrate the practicality of admix-kit through analyses of a simulated dataset. All associated code and notebooks have been made publicly accessible (https://github.com/UW-GAC/admix-kit_workflow). This ensures our results are fully reproducible and can be seamlessly deployed in a cloud platform (e.g. AnVIL). We used the AnVIL workflow to simulate *N* = 1000 admixed individuals with *M* = 174K SNPs on chromosomes 1 and 2 presented in 1000 Genomes project, using a demographic model similar to African American individuals with over 8 generations of admixture and an average ancestry proportion of 80% African and 20% European (Kidd *et al.* 2012) (ancestry proportion varies by individual). Notably, the genotype simulation took <30 minutes with scalability to a much larger number of individuals and SNPs. Using principal component analysis (PCA), we observed that individuals within the simulated dataset are positioned along a cline between individuals labeled as European and African in the 1000 Genomes reference dataset, suggesting high quality of the simulated genotype dataset (Fig. 1c and d). Allele frequencies computed within genotype segments corresponding to the respective local ancestry displayed high consistency with those computed in the reference population, indicating high preservation of MAF structure of the simulated genotype (Fig. 1e).

## 3 Discussion

Addressing the underrepresentation of admixed individuals in genetic studies is pivotal not only for scientific necessity but also as a commitment to equity. With this goal in mind, we introduce admix-kit, a comprehensive toolkit and workflow tailored for admixed populations. We anticipate that our software package and workflows will facilitate greater inclusion of admixed individuals in future genetic studies.

Development of software and methodology in genetic studies relies heavily on the use of simulated datasets. These datasets help benchmark performance and facilitate comparisons with existing software. Traditionally, simulated datasets are usually derived from publicly available reference populations. Often, these populations are selected based on a high degree of genetic similarity among individuals in the population (e.g. individuals having all four grandparents from a small geographic region.) For instance, HAPGEN2 has recently been widely used for simulating large-scale genetic datasets that mimic the LD structures of reference populations such as European, African, American, East Asian, and South Asian using data from the 1000 Genomes Project (Su *et al.* 2011, Ruan *et al.* 2022, Zhang *et al.* 2022, Miao *et al.* 2023). While these simulations can recreate datasets with similar LD as the reference populations, they cannot accurately reflect the genetic structure observed in admixed populations where ancestral segments mixing over generations (see example in Supplementary Fig. S2). Consequently, these sampling conditions are not representative of global human genetic variations. As a remedy, simulating admixture among reference populations can provide datasets that more rigorously test the performance of new software. For example, our simulation pipeline can be used to investigate factors that potentially impact accuracy of ancestry inference (including ancestry composition in reference panel, demographic model of simulated admixed population and error in inferred local ancestry) and to understand how errors in ancestry inference propagate to downstream disease mapping and prediction applications. In addition to the admixed genotype simulation provided by previously introduced admix-simu (see URLs) and haptools (Massarat *et al.* 2023), admix-kit provides a suite of methods for statistical genetic analysis of complex traits taking into account of the genetic effects heterogeneity across local ancestry backgrounds (we provide example notebooks illustrating each functionality; URLs).

Admix-kit holds significant potentials in the development of Polygenic Risk Scores (PRS). The efficacy of PRS is known to hinge on the similarity of the target population to the training population (Ding *et al.* 2023). With the PRIMED consortium working on methods to improve the performance of PRS in diverse populations, simulations will be pivotal for method evaluation (Kachuri *et al.* 2023). In this context, we expect that admix-kit will be an essential part of this effort.

## Supplementary Material

btae148_Supplementary_Data

## Data Availability

Data used in the manuscript is available from the admix-kit documentation page: https://kangchenghou.github.io/admix-kit/simulate-admix-genotype.html.
