# Supplementary material for: Admix-kit: an integrated toolkit and pipeline for genetic analyses of admixed populations
Source: Bioinformatics. 2024 Mar 15;40(4):btae148. doi: 10.1093/bioinformatics/btae148 (PMC10980565; doi:10.1093/bioinformatics/btae148)
Supplement: btae148_Supplementary_Data [file btae148_supplementary_data.zip › supp.docx]

**Supplementary Materials for**

**“Admix-kit: An Integrated Toolkit and Pipeline for Genetic Analyses of Admixed Populations”**

**
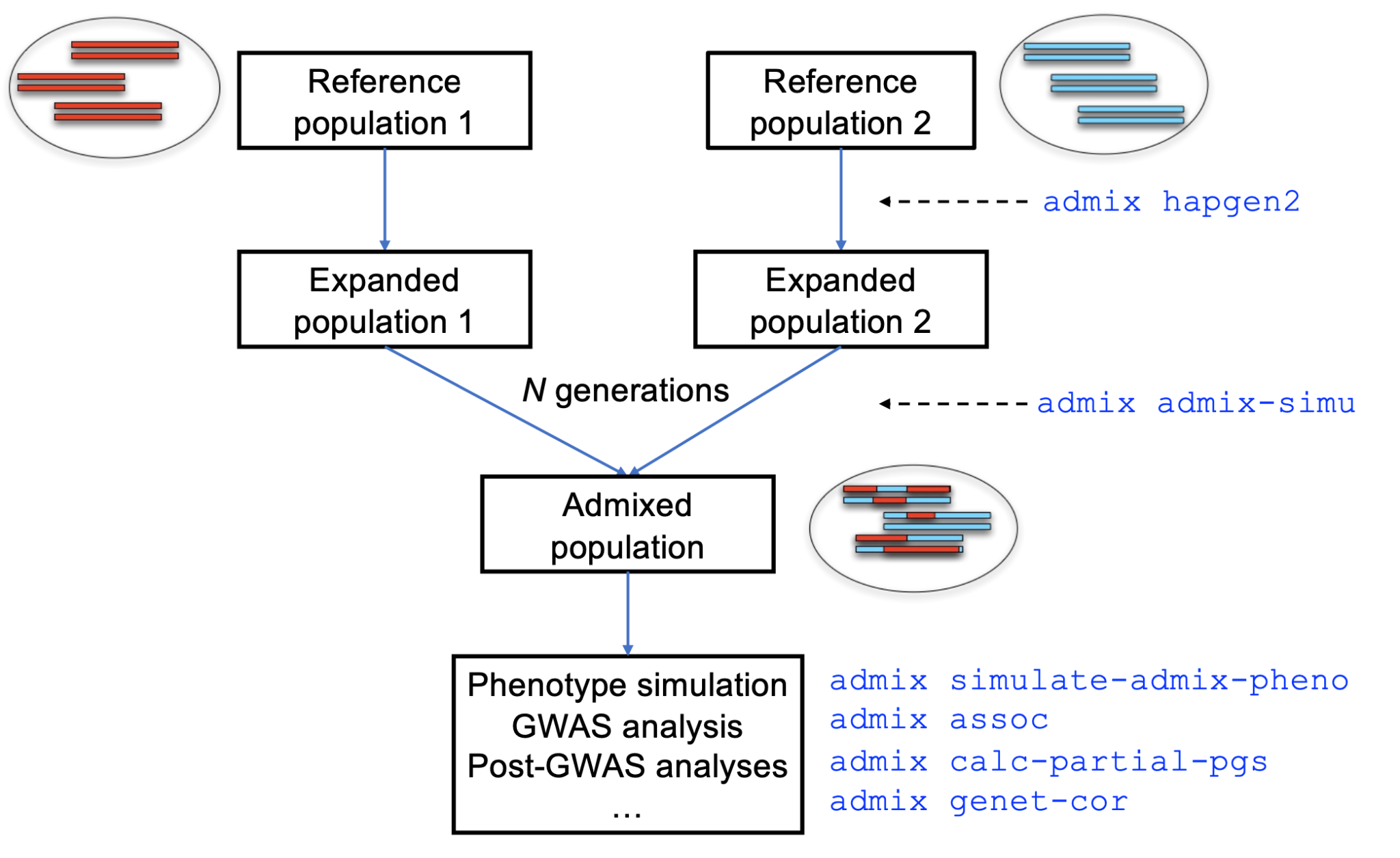
**

**
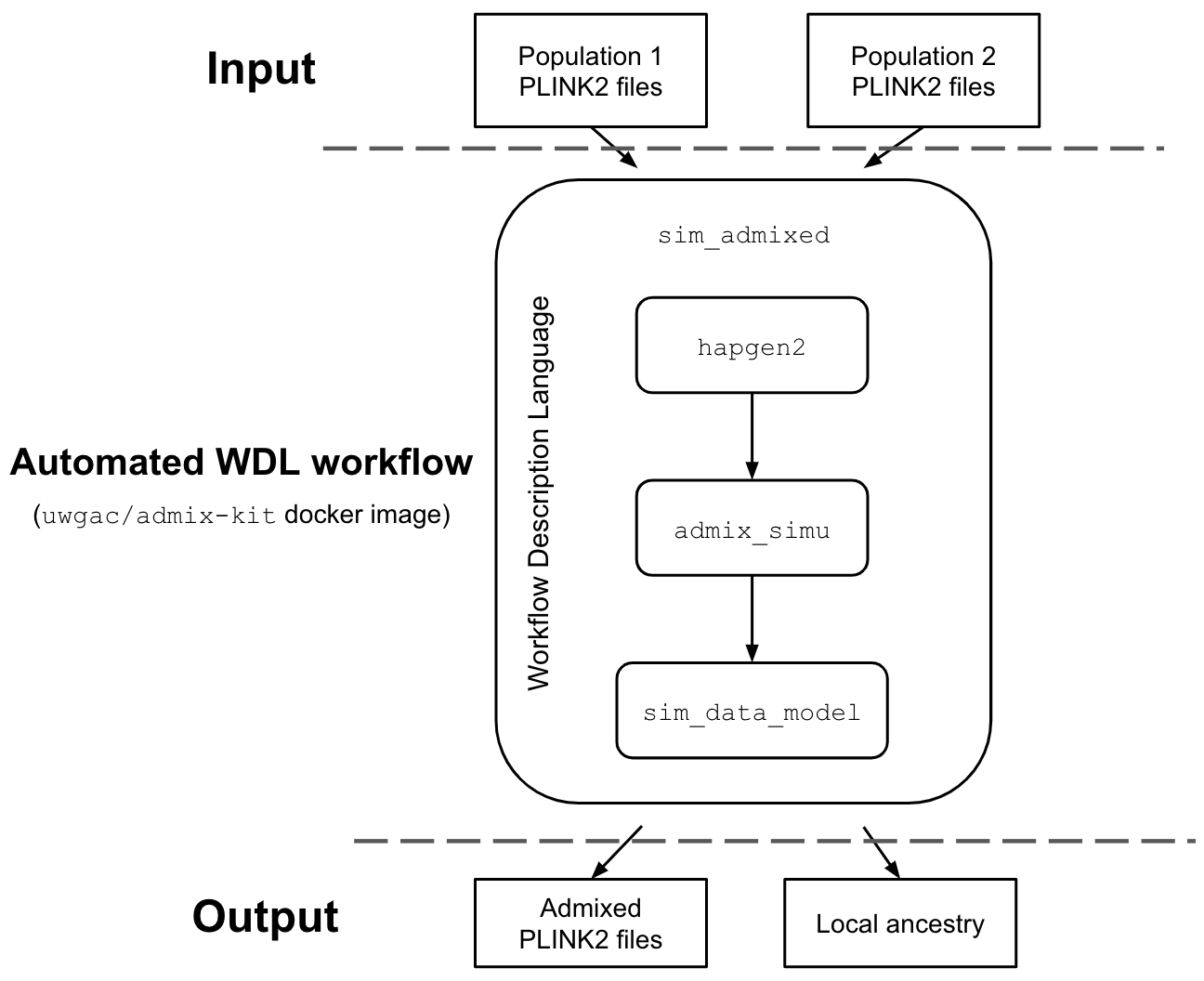
**

**Figure S1: Overview of genotype simulation for admixed populations. (upper panel)** Illustration of admixed genotype simulation and the corresponding python API. **(lower panel)** Illustration of WDL workflow.


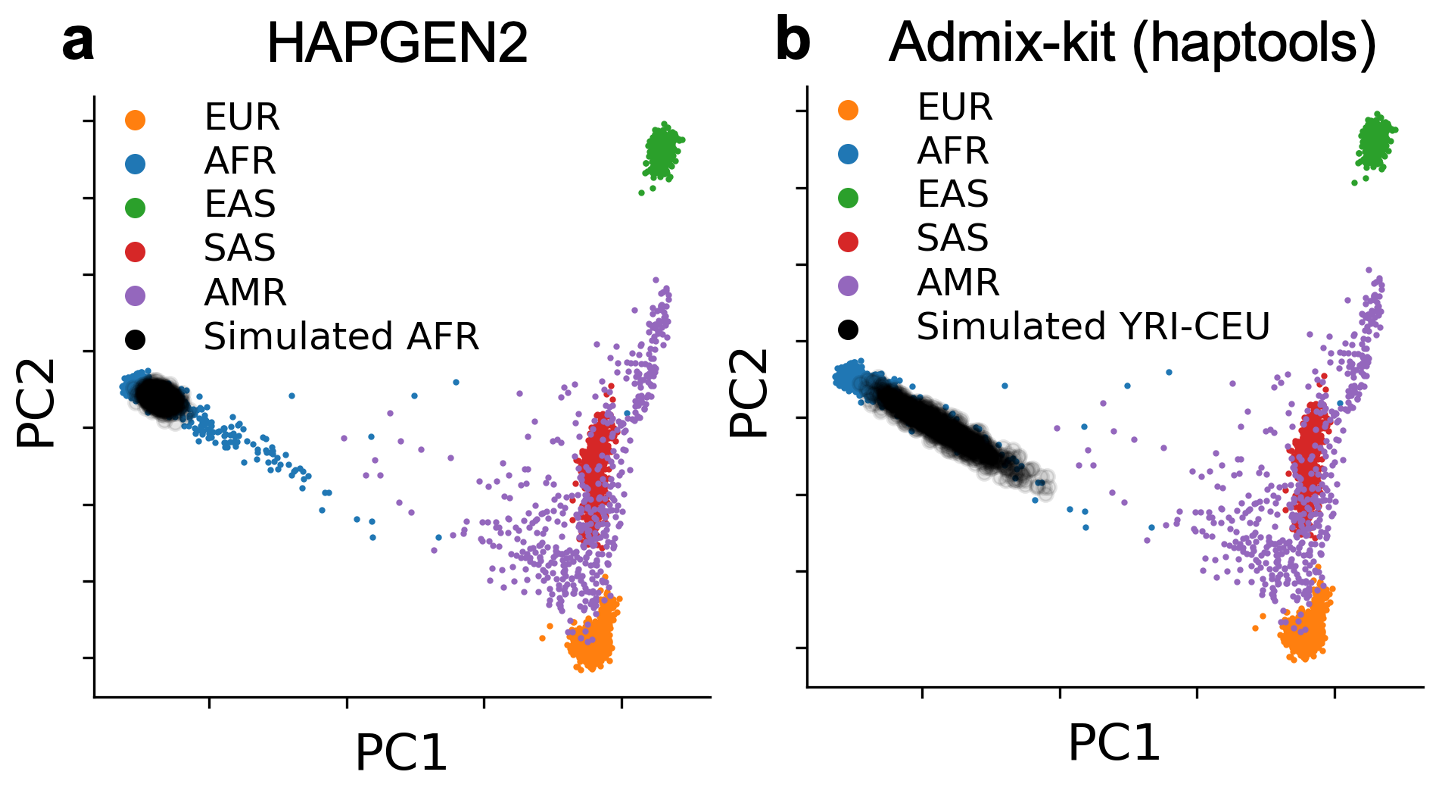


**Figure S2: comparison of simulated admixed individuals HAPGEN2 and using admix-kit (haptools)**. We attempt to use HAPGEN2 to simulate admixed individuals that are similar to AFR in 1,000 Genomes Project. We used HAPGEN2 to simulate 1,000 individuals starting with 661 AFR individuals in 1,000 Genomes. We found that the population structure and the continuous genetic ancestry variation in 1000 Genomes Project is not observed in these simulated data. Simulated individuals are relatively homogenous simulated with HAPGEN2 **(a)**, compared to those simulated with admix-kit using reference populations of YRI and CEU in 1,000 Genomes **(b)**.

**Polygenic Risk Methods in Diverse Populations (PRIMED) Consortium Methods Working Group**

Sally Adebamowo^1^, Adebowale Adeyemo^2^, Paul Auer^3^, Taoufik Bensellak^2^, Sonja Berndt^2^, Rohan Bhukar^4^, Hongyuan Cao^5^, Clinton Cario^6^, Nilanjan Chatterjee^7^, Jiawen Chen^8^, Tinashe Chikowore^9^, Ananyo Choudhury^9^, Matthew Conomos^10^, David Conti^11^, Sinead Cullina^12^, Burcu Darst^13^, Yi Ding^14^, Ruocheng Dong^15^, Rui Duan^16^, Yasmina Fakim^17^, Nora Franceschini^8^, Tian Ge^18^, Anisah W. Ghoorah^17^, Chris Gignoux^19^, Stephanie Gogarten^10^, Neil Hanchard^2^, Rachel Hanisch^2^, Michael Hauser^20^, Scott Hazelhurst^9^, Jibril Hirbo^21^, Whitney Hornsby^18^, Kangcheng Hou^14^, Xing Hua^2^, Alicia Huerta^22^, Micah Hysong^8^, Jin Jin^23^, Angad Johar^24^, Jon Judd^6^, Linda Kachuri^6^, Abram Bunya Kamiza^9^, Eimear Kenny^12^, Alyna Khan^10^, Elena Kharitonova^8^, Joohyun Kim^21^, Iain Konigsberg^19^, Charles Kooperberg^13^, Matt Kosel^24^, Iftikhar Kullo^24^, Ethan Lange^19^, Yun Li^8^, Qing Li^2^, Maria Liivrand^25^, Kirk Lohmueller^14^, Kevin Lu^21^, Ravi Mandla^4^, Alisa Manning^4^, Iman Martin^2^, Alicia Martin^4^, Shannon McDonnell^24^, Leah Mechanic^2^, Josep Mercader^4^, Rachel Mester^14^, Maggie Ng^21^, Kevin Nguyen^1^, Kristján Norland^24^, Franklin Ockerman^8^, Loes Olde Loohuis^14^, Ebuka Onyenobi^1^, Bogdan Pasaniuc^14^, Aniruddh Patel^4^, Ella Petter^14^, Kenneth Rice^10^, Joseph Rothstein^12^, Bryce Rowan^12^, Robb Rowley^2^, Yunfeng Ruan^4^, Sriram Sankararaman^14^, Ambra Sartori^7^, Dan Schaid^24^, Ruhollah Shemirani^12^, Jonathan Shortt^19^, Xueling Sim^26^, Johanna L. Smith^24^, Maggie Stanislawski^19^, Daniel Stram^11^, Quan Sun^8^, Bamidele Tayo^27^, Buu Truong^4^, Kristin Tsuo^4^, Sarah Urbut^18^, Ying Wang^4^, Wallace Minxian Wang^4^, Riley Wilson^2^, John Witte^6^, Genevieve Wojcik^7^, Jingning Zhang^7^, Ruyue Zhang^8^, Haoyu Zhang^2^, Yuji Zhang^1^, Michael Zhong^1^, Laura Zhou^8^

^1^University of Maryland Baltimore , Baltimore, MD, United States, ^2^National Institutes of Health, Bethesda, MD, United States, ^3^Medical College of Wisconsin, Milwaukee, WI, United States, ^4^Broad Institute, Cambridge, MA, United States, ^5^Florida State University, Tallahassee, FL, United States, ^6^Stanford University, Stanford, CA, United States, ^7^Johns Hopkins University, Baltimore, MD, United States, ^8^University of North Carolina at Chapel Hill, Chapel Hill, NC, United States, ^9^University of the Witwatersrand, Johannesburg South Africa, ^10^University of Washington, Seattle, WA, United States, ^11^University of Southern California, Los Angeles, CA, United States, ^12^Mount Sinai, New York City, NY, United States, ^13^Fred Hutchinson Cancer Center, Seattle, WA, United States, ^14^University of California Los Angeles, Los Angeles, CA, United States, ^15^University of Wisconsin Milwaukee, Milwaukee, WI, United States, ^16^Harvard, Cambridge, MA, United States, ^17^University of Mauritius, Réduit Mauritius, ^18^Massachusetts General Hospital, Boston, MA, United States, ^19^University of Colorado, Aurora, CO, United States, ^20^Duke University, Durham, NC, United States, ^21^Vanderbilt University Medical Center, Nashville, TN, United States, ^22^Instituto Nacional de Ciencias Médicas y Nutrición Salvador Zubiran, Mexico City, CDMX, Mexico, ^23^University of Pennsylvania, Philadelphia, PA, United States, ^24^Mayo Clinic, Rochester, MN, United States, ^25^Genevia Technologies, Tampere Finland, ^26^National University of Singapore, Singapore, ^27^Loyola University of Chicago, Chicago, IL, United States
